# Procaine and saline have similar effects on articular cartilage and synovium in rat knee

**DOI:** 10.1186/s12871-018-0513-9

**Published:** 2018-05-09

**Authors:** Aysun Ankay Yilbas, Basak Akca, Berkem Buyukakkus, Elham Bahador Zirh, Dilara Zeybek, Filiz Uzumcugil, Fatma Saricaoglu

**Affiliations:** 1 Department of Anesthesiology and Reanimation, Hacettepe University, Faculty of Medicine, Sıhhiye, Ankara, Turkey; 2 Department of Histology and Embryology, Hacettepe University, Faculty of Medicine, Ankara, Turkey

**Keywords:** Procaine, Rats, Knee joint, Intra-articular injections, Local anaesthetics

## Abstract

**Background:**

Intra-articular local anaesthetics are widely used for providing postoperative analgesia and decreasing the need for opioids. Procaine has proven positive effects in carpal tunnel syndrome and chondromalacia patella. However, the effect of procaine on articular cartilage has not yet been studied. The aim of this study was to evaluate the effects of intra-articular procaine injection on the articular cartilage and the synovium.

**Methods:**

Twenty adult Sprague-Dawley rats were enrolled in the study. After providing anaesthesia and aseptic conditions, 0.25 ml of 10% procaine was injected to the right knee joint, and 0.25 ml of normal saline (as control group) was injected to the left knee joint. Knee joint samples were obtained from four rats in each group after appropriate euthanasia on days 1, 2, 7, 14 and 21. The histological sections of the articular and periarticular regions and the synovium were evaluated by two histologists, and inflammatory changes were graded according to a five-point scale in a blinded manner. The apoptosis of chondrocytes was determined by the caspase-3 indirect immunoperoxidase method.

**Results:**

There were no significant differences in inflammation between procaine and saline groups at any of the time intervals. Slight inflammatory infiltration due to injection was seen in both groups on the 1st day. Haemorrhage was observed in both groups at days 1 and 2, and the difference between groups was not found to be significant. No significant difference was detected in the percentage of apoptotic chondrocytes between groups at any of the time intervals.

**Conclusions:**

Injection of procaine seems safe to use intra-articularly based on this in vivo study on rat knee cartilage. However, further studies investigating both the analgesic and histopathological effects of procaine on damaged articular cartilage and synovium models are needed.

## Background

Intra-articular local anaesthetics are widely used for providing postoperative analgesia and decreasing the need for opioids and side effects related to their usage. In addition to single injections at the end of arthroscopic surgery or in cases of chronic inflammatory pain, infusion pumps are commonly used for longer duration of effect [1]. However, recent in vitro and in vivo studies showed chondrotoxic effects of some widely used intra-articular local anaesthetics, especially bupivacaine and lidocaine [1–3].

Procaine, which is one of the most widely used local anaesthetics in neural therapy [4–6], has proven positive effects in carpal tunnel syndrome and chondromalacia patella [6, 7]. In vitro studies also showed that procaine and its metabolite diethylaminoethanol (DEAE) decrease the formation of free oxygen radicals in leucocytes [8]. Although the mechanisms are not totally clear, procaine has inhibitory effects on the release of inflammatory mediators such as eicosanoids, which have a vital role in pathologic responses to tissue damage and regulation of physiological organ function [9]. Therefore, we hypothesized that procaine could be an alternative local anaesthetic to use intra-articularly with potential anti-inflammatory effects.

However, the effects of procaine on articular cartilage and the synovium have not yet been studied. The aim of this study was to evaluate the histopathological effects of intra-articular procaine injection on the articular cartilage and the synovium.

## Methods

The study was conducted in accordance with the European Union Strategy for the Protection and Welfare of Animals. Following approval from the Animal Care and Use Ethical Committee of Hacettepe University (approval number: 52338575-05, 03.02.2015), 20 adult male Sprague-Dawley rats were enrolled in the study. The study was conducted in the animal laboratory of a tertiary university hospital, and all animals were kept under optimum conditions (separate cages enough to provide free activity, appropriately controlled temperature of 22 ± 2 °C, humidity 50-55%, 12 h light/12 h dark) and received food and water ad libitum.

**Fig. 1:**
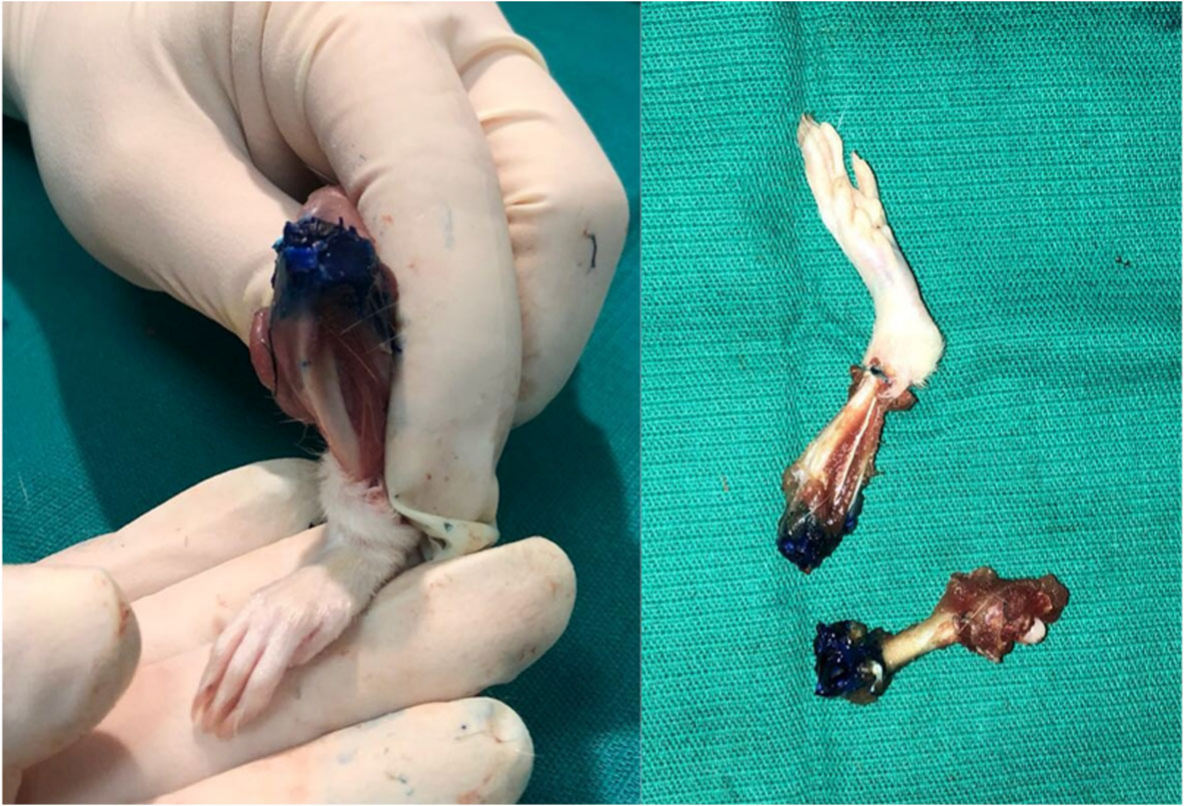
The macroscopic view of the knee joint following methylene blue injection

The rats were anaesthetized with 80 mg/kg intraperitoneal ketamine. After providing aseptic conditions, 0.25 ml of 10% procaine was injected to the right knee joint, and 0.25 ml of normal saline (as control group) was injected to the left knee joint of each rat. All intra-articular injections were performed, in dorsal recumbent position, by the same person to avoid possible bias due to personal differences in technique. A 26-gauge 1-ml insulin syringe was used. Following palpation of the patella, the needle was inserted through the frontolateral side of the infrapatellar ligament (slightly below the patella) into the knee joint cavity, gently and slowly until the needle was felt to pop into the cavity, and 0.25 ml of solution was injected with verification of no soft tissue swelling and no feeling of resistance. Before starting the study, the technique was tested and proven by injecting 0.25 ml of methylene blue to both knees of an appropriately anaesthetized rat. The macroscopic examination of both knees showed perfectly dyed knee joint cavities (Fig. 1).

After injections, animals were again placed in their separate cages. Knee joint samples were obtained from four rats in each group after appropriate euthanasia (120 mg/kg intraperitoneal ketamine followed by cervical dislocation) on days 1, 2, 7, 14 and 21.

The knee joints were fixed with 10% buffered formalin for 2 weeks and decalcified in “De Castro” solution for 6 weeks at room temperature. Then, decalcified samples were processed for routine light microscopy. Paraffin blocks were prepared, and 5-μm-thick sections were cut and stained with haematoxylin-eosin and Alcian blue.

The apoptotic cells were determined by caspase-3 immunoreactivity. Briefly, the sections were incubated at 60 °C overnight and then cleared in xylene for 15 min. After rehydrating through a decreasing series of alcohols, sections were washed with 0.01 M phosphate buffer saline (PBS) at pH 7.4 for 5 min. Heat-mediated antigen retrieval was performed in Tris/EDTA buffer (pH 9.0) by pressure cooker for 3 min. Sections were cooled and washed with PBS three times for 5 min. Endogenous peroxidase was blocked by incubation in 10% H2O2 in PBS for 5 min at 4 °C. Unspecific binding was blocked using goat serum at a dilution of 1:10 for 30 min at room temperature. Then, sections were incubated with anti-rabbit caspase-3 (ab184787, Abcam) primary antibody for 1 h at room temperature. After washing 3 times for 5 min with PBS, sections were incubated with goat anti-rabbit secondary antibody (ab97051, Abcam) for 30 min. After washing in PBS, peroxidase activity was revealed by incubation with DAB for 5 min and counterstaining with Mayer’s haematoxylin. After washing with tap water, sections were dehydrated through graded alcohols and cleared in xylene before mounting with Entellan. Negative-control experiments were performed by omitting incubation with the primary antibody. The sections were examined and photographed by a light microscope (Leica DM 6000B) with a DFC490 digital camera (Leica, Wetzlar, Germany).

In the histological sections of the knee joints, the surface of the cartilage, cartilage zones (tangential, intermediate, radial and calcified zone), the synovium and soft connective tissue beyond the synovium were evaluated by two histologists who were blinded to the joint treatment groups. The inflammatory changes were graded in four fields per section and four sections per animal according to a five-point scale, based on a similar study of Erden et al. [10].

- Grade 0- no inflammation
- Grade 1- minimal inflammation: mild congestion and oedema
- Grade 2- mild inflammation: erosion of joint surface, congestion and oedema, and small number of neutrophils
- Grade 3- moderate inflammation: neutrophils and macrophages, and synoviocyte hyperplasia
- Grade 4- severe inflammation: neutrophils and macrophages, synoviocyte hyperplasia, and fibrin exudation

Microscopic haemorrhage was also graded according to a five-point scale (0 = no haemorrhage, 1 = minimal, 2 = moderate, 3 = severe and 4 = very severe).

The percentage of chondrocytes staining positive for caspase-3 was calculated by counting the number of positively and negatively stained cells in each cartilage zone, including superficial, intermediate and deep zones, in 5 areas in each zone of each sample [11].

### Sample size estimation

The primary outcome for the study was the inflammation scores. A total sample size of 20 (4 per group) was required to detect at least 65% difference in inflammation scores between saline and procaine groups on day 2 with a power of 80% at the 5% significance level. To avoid unnecessary laboratory animal sacrifice, one knee of the same rat was used as the study group, while the other knee was used as the control group. Sample size estimation was performed by using G*Power (Franz Faul, Universität Kiel, Kiel, Germany) version 3.0.10.

SPSS software version 21.0 (SPSS Inc., Chicago, IL) was used for data analysis. As the number of rats in each group was four, data were considered to be non-normally distributed and Mann-Whitney U test was used to compare the differences between procaine and saline groups at all time intervals. The median (minimum-maximum) was used for data description. *p* < 0.05 was considered significant.

## Results

**Fig. 2:**
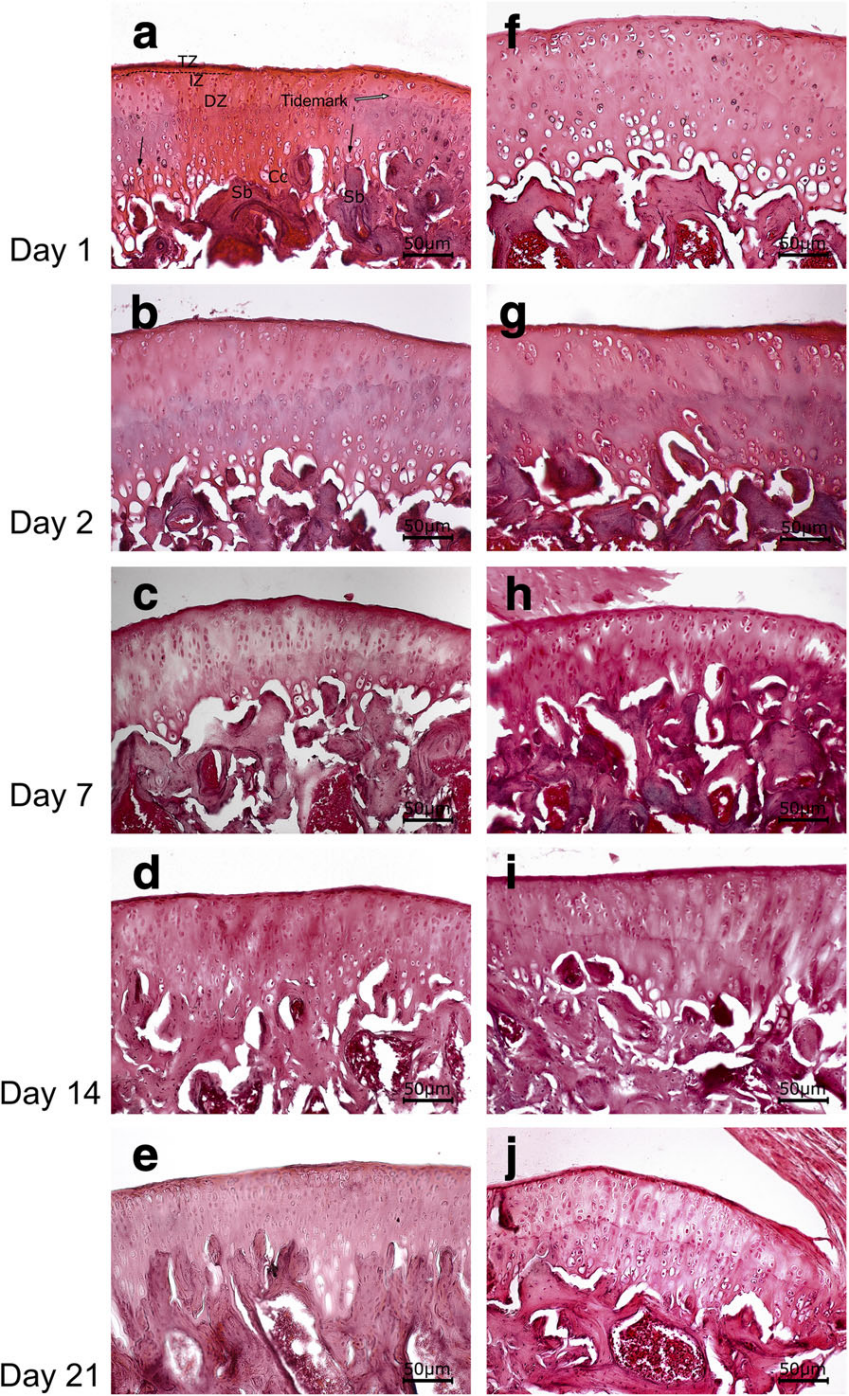
The articular cartilage of the knee joints. Intact cartilage surfaces without erosion at all time intervals in both saline (**a**–**e**) and procaine (**f**–**j**) administered groups. TZ: Tangential zone, IZ: Intermediate zone, RZ: Radial zone, Cc: Calcified cartilage, SB: Subchondral bone, White arrows: Tidemark, Black arrow: Hypertrophied chondrocytes. **a**–**e** show saline-administered groups, and **f**–**j** show procaine-administered groups. Haematoxylin and eosin, Scale bar: 50 μm

The mean weight of rats was 378.25 ± 35.55 g (min 310 – max 450 g). A total of 40 rat knee joints were examined, and no macroscopic haematomas were observed in any of the knee samples. Normal histological structure of the articular cartilage with consequent zones was observed in both groups. The flattened chondrocytes were observed in the superficial (tangential) zone under the articular surface. Randomly distributed round chondrocytes were observed in the intermediate zone. The round chondrocytes arranged in columns perpendicular to the free articular surface were examined in the radial zone. Tidemark was distinguished as a smooth, undulating basophilic line. The hypertrophy of chondrocytes, calcified cartilage and subchondral bone was observed. The surface of the articular cartilage was intact, and cartilage erosion, joint destruction and pannus formation were not seen at any time interval in both groups (Fig. 2).

**Fig. 3:**
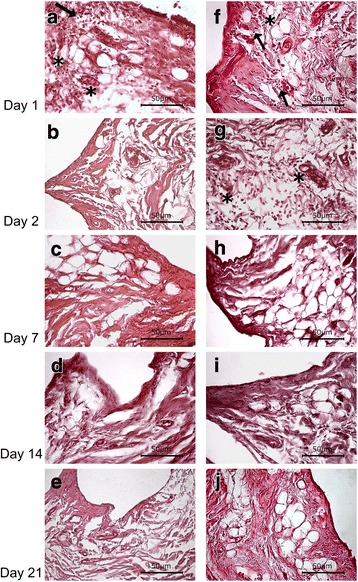
The synovium of knee joints. Slight inflammatory infiltration (arrow) and haemorrhage (asterisk) was observed in the saline group at day 1 and in the procaine-administered group at days 1 and 2. No inflammation was observed in the synovium at days 7, 14 and 21. Synoviocytes surrounding the connective tissue were seen. **a**–**e** show saline-administered groups, and **f**–**j** show procaine-administered groups. Haematoxylin and eosin, Scale bar: 50 μm

A single row of synoviocytes was lining over the highly vascularized connective tissue in the saline group. Slight inflammatory infiltration and prominent haemorrhage were observed in this group at day 1. Inflammatory grade was 1 at day 1 and 0 at days 2, 7, 14, and 21 (Fig. 3a–e).

Mild congestion and oedema were examined in the procaine group at days 1 and 2. Different degrees of haemorrhage were also observed in the procaine group at days 1 and 2. Compared to the saline group, haemorrhage score differences at days 1 and 2 were not significant in the procaine group (*p* = 1.000 at day 1, *p* = 0.057 at day 2). There was slight inflammatory infiltration in certain regions of the synovium at days 1 and 2 after procaine administration. Inflammatory grade was 1 at days 1 and 2 (Figs. 2g and 3f). There was no infiltration in the synovium at days 7, 14 and 21 in the procaine-administered group (Fig. 3h–j). The synovial membrane was of normal thickness with synoviocytes surrounding the connective tissue composed of collagen fibres; adipocytes were observed in this group, and there was no synovitis at days 7, 14, and 21 (Fig. 3). Table 1 shows results of the histopathological evaluation of each knee joint.

**Table 1:** Comparison of histopathological evaluation of inflammatory changes and hemorrhage between groups

	Group procaine	Group saline	*p*
Inflammatory changes			
1st day	1 (1-1)	0.5 (0-1)	0.343
2nd day	1 (1-1)	0 (0-1)	0.114
7th day	0 (0-0)	0 (0-1)	0.686
14th day	0 (0-1)	0 (0-0)	0.686
21st day	0 (0-0)	0 (0-0)	1.000
Hemorrhage			
1st day	1.5 (1-2)	1.5 (0-3)	1.000
2nd day	3 (1-3)	0 (0-2)	0.057
7th day	0 (0-1)	0 (0-1)	1.000
14th day	0.5 (0-1)	0 (0-1)	0.686
21st day	0 (0-0)	0 (0-0)	1.000

The data was expressed as median (minimum-maximum)

**Fig. 4:**
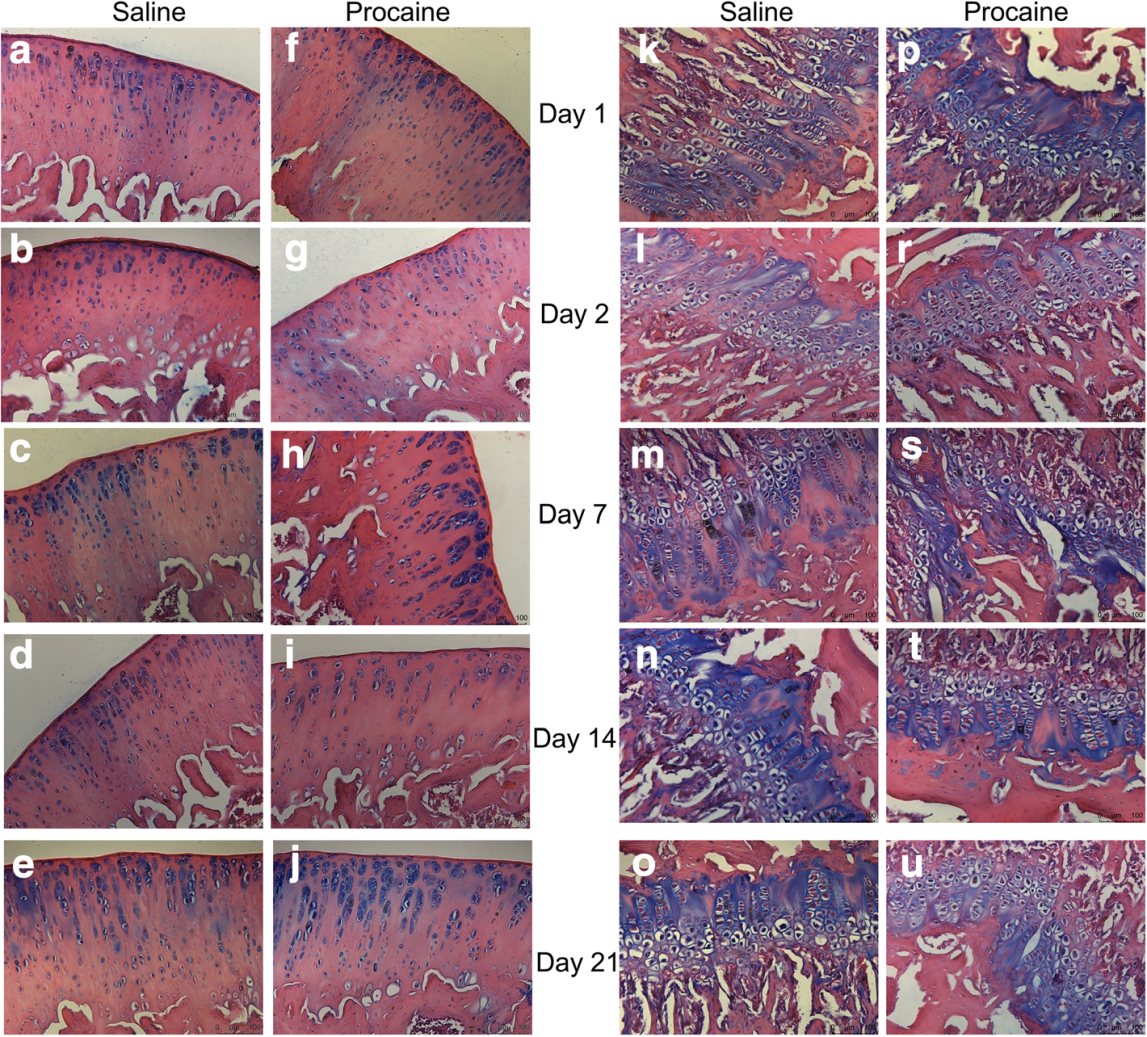
The articular cartilage and epiphyseal plates of experimental groups. The extracellular matrix showed similar glycosaminoglycan content in saline and procaine groups both in the articular cartilage and epiphyseal plates. **a**–**e** and **f**–**j** show the articular cartilage of knee joints in saline and procaine, respectively. **k**–**o** and **p**–**u** show the epiphyseal plates in saline and procaine, respectively. Alcian blue-haematoxylin-eosin, Scale bar: 100 μm

Alcian blue staining was used for the evaluation of proteoglycan in the matrix. The staining of saline and procaine groups was similar at all time intervals, and there was no reduction in the content of glycosaminoglycan in the matrix with procaine administration (Fig. 4).

**Fig. 5:**
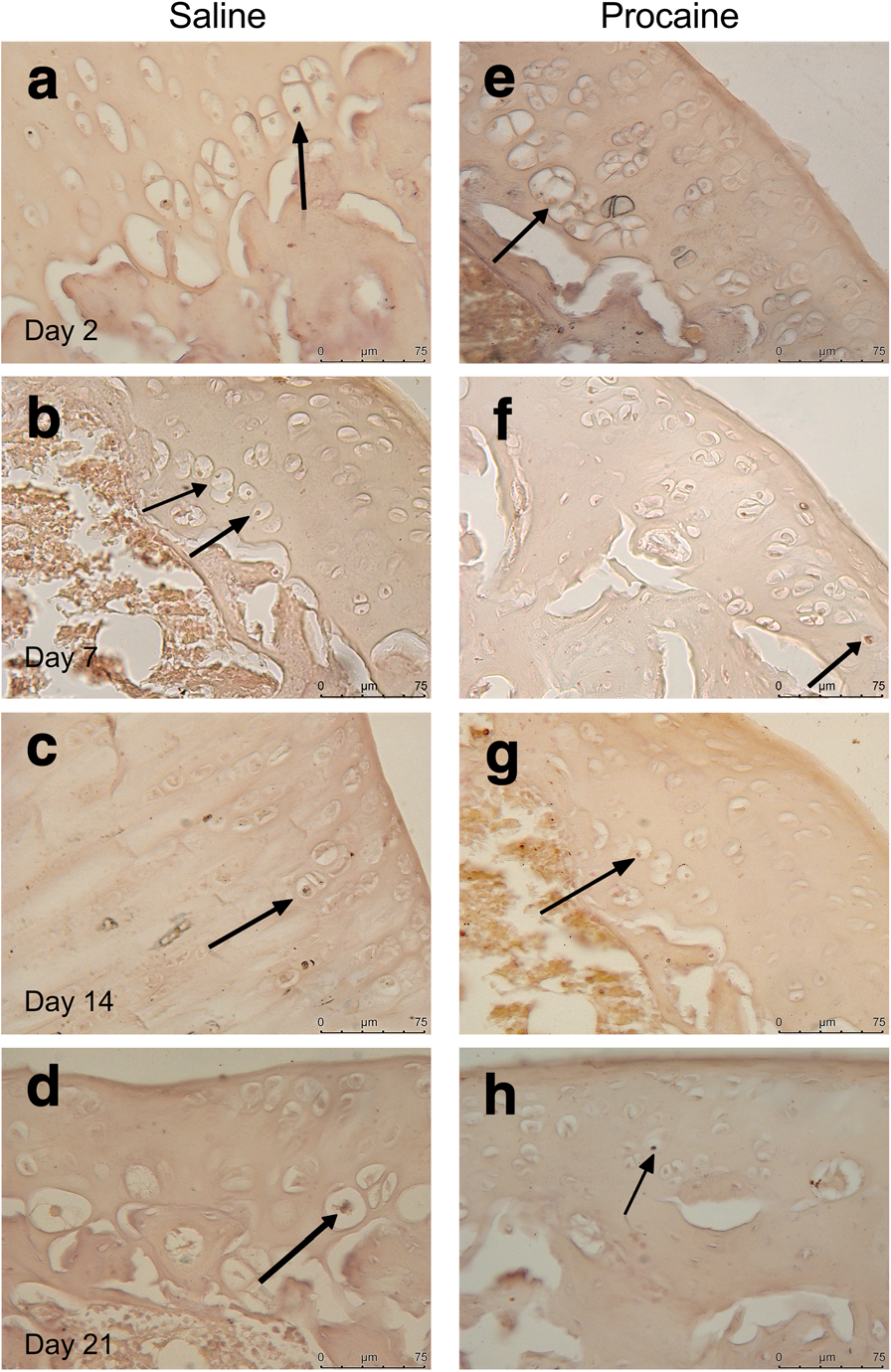
The caspase-3 immunoreactivity in the articular cartilage of knee joints. The caspase-3 positive chondrocytes (arrow) in both groups. **a**–**d** show saline-administered groups, and **e**–**h** show procaine-administered groups. Scale bar: 50 μm

The percentage of caspase-3 positive chondrocytes was similar in both saline and procaine groups at all time intervals. No significant difference was detected in the percentage of apoptotic chondrocytes between groups at any of the time intervals (Fig. 5, Table 2).

**Table 2:** Caspase-3 immune reactivity in zones of articular cartilage

Caspase-3 immune reactivity in zones of articular cartilage (% of apoptotic cells in each zone)			
	Group procaine	Group saline	*p*
1st day			
Superficial zone	8.04 ± 3.90	9.69 ± 3.93	0.360
Intermediate zone	9.54 ± 4.41	9.68 ± 5.05	0.948
Deep zone	10.32 ± 3.25	10.69 ± 4.15	0.828
2nd day			
Superficial zone	8.54 ± 4.13	8.98 ± 3.12	0.791
Intermediate zone	10.46 ± 2.93	10.73 ± 3.46	0.852
Deep zone	10.70 ± 3.01	10.22 ± 2.94	0.722
7th day			
Superficial zone	8.22 ± 4.07	10.65 ± 3.69	0.179
Intermediate zone	8.11 ± 3.05	9.75 ± 3.61	0.286
Deep zone	10.37 ± 3.60	10.16 ± 3.29	0.897
14th day			
Superficial zone	8.27 ± 3.92	10.06 ± 6.00	0.439
Intermediate zone	9.25 ± 2.91	10.69 ± 4.07	0.377
Deep zone	10.55 ± 4.37	9.39 ± 2.80	0.491
21st day			
Superficial zone	9.41 ± 3.99	9.61 ± 5.06	0.924
Intermediate zone	9.43 ± 3.52	10.02 ± 3.52	0.712
Deep zone	10.04 ± 3.01	10.37 ± 3.69	0.828

Data represents the mean percentage of apoptotic chondrocytes ± standard deviation

## Discussion

Procaine and saline groups were found similar in terms of inflammation and chondrotoxicity when injected intra-articularly at all time intervals in our study.

Systemic toxicity of intra-articularly used local anaesthetics is rare due to minimal distribution, but chondrotoxicity is still a debate. A single injection of various concentrations of lidocaine caused significant decrease in chondrocyte viability in both human and animal cell cultures, and this time a dose-dependent effect was associated with mitochondrial injury [12–16]. Several studies also showed that exposure to bupivacaine, especially in concentrations of 0.5%, was leading to a decrease in proteoglycan synthesis and chondrocyte viability [1–3, 17]. In Chu et al.’s study, a nearly 50% decrease in cell density was observed even after 6 months from a single injection of 0.5% bupivacaine into the stifle joints of rats [2]. These findings suggest that intra-articular local anaesthetics, especially when used as infusion, may be related to increased risk of postartroscopic chondrolysis and early osteoarthritis [1]. Higher concentrations, low pH (< 5), infusion pump usage for longer lasting effect and the use of epinephrine, which decreases pH, are among the factors related to increased chondrotoxicity [1, 10]. Dogan et al. [18] also conducted an experimental study on rabbit knees and found that compared to saline in joint cartilage and the synovium, bupivacaine and neostigmine injection histopathologically caused an increase in inflammation, inflammatory cell infiltration, synovial hyperplasia, and hypertrophy. The molecular mechanism of chondrotoxicity could be explained by the influence of local anaesthetics on potassium, calcium and sodium channels leading to mitochondrial dysfunction and cell death [19].

Ropivacaine and levobupicaine seem to be safer. Dragoo et al. [16] did not find any signs of increased chondrotoxicity following exposure of in vitro human chondrocytes to 0.25% bupivacaine and 0.5% ropivacaine. Although the analgesic efficacy of intra-articular levobupivacaine has been shown in several human studies, we could find only two histopathological studies evaluating the chondrotoxicity of levobupivacaine in the literature [10, 20]. The design of these experimental animal studies, which were also conducted at our university, was very similar to our study and thus has the same limitations of not being directly applicable to human tissue. In Erden et al.’s study [10], compared to saline injected knees, the degree of inflammation changes on the 1st day were significantly higher in the levobupivacaine-injected rat knees, but both groups were similar from the 7th to 21st day. The study group decided that levobupicaine seemed to be safe. In contrast, the study of Kurkcuoglu et al. [20] comparing the effects of intraarticular levobupivacaine and bupivacaine, again in rat knees, demonstrated signs of mild to moderate inflammation still on the 7th, 14th and 21st days of injection and signs of fibrosis during recovery in both groups. Additionally, there was one rat with severe inflammation on the 7th day due to levobupicaine. In our study, we did not see any inflammation more than mild congestion and oedema in any of the groups, and there was nearly no inflammation after the 7th day. Although haemorrhage was thought to be more prominent in the procaine group on the 2nd day, we could not find any significant difference (*p* = 0.057). This result may be different in a larger sample group. We could not compare our results regarding haemorrhage with other study findings because none of the similar studies in the literature did grade microscopic haemorrhage before. The slight inflammatory infiltration and haemorrhage seen in both groups on the first 2 days were thought to be due to the needle insertion itself.

Some local anaesthetics, including procaine, seem to have significant anti-inflammatory properties via inhibiting leucocyte adhesion, granulocyte phagocytosis and release of inflammatory mediators and oxygen free radicals. These anaesthetics have been used successfully in conditions such as burn injuries, ulcerative colitis, acute pancreatitis and arthritis [9]. Procaine and its metabolite, diethylaminoethanol, have been proven to decrease free oxygen radicals and antigen expression in both experimental rabbit and human tissue studies [8, 21]. Karadas et al. [22] also found procaine similar to corticosteroids to reduce the pain and improve electrophysiological findings in carpal tunnel syndrome patients. However, our study is the first to evaluate the histopathological effects of intra-articular procaine, an old drug gaining popularity again due to its anti-inflammatory effects. This study was planned, as a pilot study of further experimental studies investigating whether intra-articular procaine could have a role in controlling excessive inflammatory response without causing any chondrotoxicity.

The exact mechanism of chondrotoxicity of local anaesthetics has not been totally explained; however, mitochondrial dysfunction, secondary to the blockade of potassium, sodium and calcium channels, seems to be the leading probable cause. Mitochondrial dysfunction, followed by decreased chondrocyte metabolism and increased apoptosis, could be thought to cause gross cartilage degradation at the end, related to the concentration and the chronic use of the local anaesthetic [1, 16]. In addition to anti-inflammatory properties, procaine has also been shown to facilitate oxygen transport towards the mitochondrial matrix of the rat brain [23]. These effects might address the question of whether procaine could be an alternative intra-articular local anaesthetic without causing any chondrotoxicity. However, the mitochondrial effects of procaine in cartilage tissue have not been studied before.

There are some important limitations of our study. First, the effect of procaine in the rat knee joint may not directly reflect the effect in human tissue. The healthy knee joint also does not actually mimic the clinical situation of the injured knee joint, which we face in our daily medical practice. Intact perichondrium has proven to be protective [24]. The second limitation is that our study was designed to assess the histopathological changes following just a single injection of a fixed dose. We could not find any data in the literature regarding the dosage of intra-articular procaine in rats. As the chondrotoxicity of many local anaesthetics has been proven to be dose-dependent [1], we decided to use procaine at a high dose of 10% in order to increase the possibility of detecting any chondrotoxic effects. The short-acting analgesic effect of procaine may be another limitation in clinical usage. Nevertheless, it is also known that procaine shows its effect at the site of injection due to its limited diffusibility [25]. Although the sample size was determined with an appropriate power analysis (80% power, α = 0.05) regarding the inflammation scores, the small group sample could also be conceivable as a limitation, especially regarding the results related to haemorrhage. The groups were found similar according to haemorrhage scores in the current study; however, haemorrhage was thought to be more prominent, especially on the 2nd day in the procaine group (*p* = 0.057). This difference might possibly be both clinically and statistically significant if studied with a larger sample size.

## Conclusions

Injection of procaine seems safe to use intra-articularly based on this in vivo study on rat knee cartilage. However, previous controversial results in different studies with other local anaesthetics, such as levobupivacaine, should lead clinicians to be cautious about the effects of intra-articular injections. Further studies investigating both the analgesic and histopathological effects of procaine on damaged articular cartilage and synovium models are needed.

## Abbreviation

DEAE: Diethylaminoethanol
